# Climatic modification effects on the association between PM1 and lung cancer incidence in China

**DOI:** 10.1186/s12889-021-10912-8

**Published:** 2021-05-07

**Authors:** Huagui Guo, Xin Li, Weifeng Li, Jiansheng Wu, Siying Wang, Jing Wei

**Affiliations:** 1 School of Architecture and Urban-rural Planning, Fuzhou University, Fuzhou, 350108 China; 2 Department of Architecture and Civil Engineering, City University of Hong Kong, Hongkong, China; 3 Department of Urban Planning and Design, The University of Hong Kong, Hongkong, China; 4 Shenzhen Institute of Research and Innovation, The University of Hong Kong, Shenzhen, 518057 People’s Republic of China; 5 Key Laboratory for Urban Habitat Environmental Science and Technology, Shenzhen Graduate School, Peking University, Shenzhen, 518055 People’s Republic of China; 6 Key Laboratory for Earth Surface Processes, Ministry of Education, College of Urban and Environmental Sciences, Peking University, Beijing, 100871 People’s Republic of China; 7 Department of Atmospheric and Oceanic Science, Earth System Science Interdisciplinary Center, University of Maryland, College Park, MD USA

**Keywords:** Modification effects, Climatic factors, PM1 pollution, Lung cancer incidence

## Abstract

**Background:**

Nationwide studies that examine climatic modification effects on the association between air pollution and health outcome are limited in developing countries. Moreover, few studies focus on PM1 pollution despite its greater health effect.

**Objectives:**

This study aims to determine the modification effects of climatic factors on the associations between PM1 and the incidence rates of lung cancer for males and females in China.

**Methods:**

We conducted a nationwide analysis in 345 Chinese counties (districts) from 2014 to 2015. Mean air temperature and relative humidity over the study period were used as the proxies of climatic conditions. In terms of the multivariable linear regression model, we examined climatic modification effects in the stratified and combined datasets according to the three-category and binary divisions of climatic factors. Moreover, we performed three sensitivity analyses to test the robustness of climatic modification effects.

**Results:**

We found a stronger association between PM1 and the incidence rate of male lung cancer in counties with high levels of air temperature or relative humidity. If there is a 10 μg/m^3^ shift in PM1, then the change in male incidence rate relative to its mean was higher by 4.39% (95% CI: 2.19, 6.58%) and 8.37% (95% CI: 5.18, 11.56%) in the middle and high temperature groups than in the low temperature group, respectively. The findings of climatic modification effects were robust in the three sensitivity analyses. No significant modification effect was discovered for female incidence rate.

**Conclusions:**

Male residents in high temperature or humidity counties suffer from a larger effect of PM1 on the incidence rate of lung cancer in China. Future research on air pollution-related health impact assessment should consider the differential air pollution effects across different climatic conditions.

**Supplementary Information:**

The online version contains supplementary material available at 10.1186/s12889-021-10912-8.

## Introduction

Severe air pollution, especially particular matter pollution, has become a global concern. A large number of studies have reported the adverse effect of air pollution on human health [1, 2]. Nonetheless, there is substantial heterogeneity in the estimates of air pollution effects among these studies. The differential estimates may partly result from the potential effect modifiers including meteorological conditions [3, 4], baseline climates [5, 6] and socioeconomic statuses [7–9]. A better understanding of such effect modifiers is not only an essential methodological issue in air pollution studies, but also important to understand the underlying etiological pathways of air pollutions. Therefore, there is a growing interest recently to identify these modifiers. Despite a few studies investigating temperature modification effects [10–13], however, whether climatic factors modify the effect of air pollution on human health is not yet well understood in China.

The relationship between PM and lung cancer diseases has been well documented. The mechanism of how air pollution (including PMs) causes lung cancer diseases has been well specified in the Volume 109 of the International Agency for Research on Cancer Monographs on the Evaluation of Carcinogenic Risks to Humans [2]. Empirically, several studies have reported the adverse effects of PM on the morbidity and mortality of lung cancer [1, 14, 15]. However, studies examining the effect of PM1 on health outcome (including lung cancer) are quite limited despite the larger effect of PM1 (compared to those of the two other prominent PMs in Chinese cities, i.e. PM2.5 and PM10) reported in Chinses cities [16, 17].

Regarding climatic modification effects on air pollution-health outcome association, several explanations have been proposed although the mechanisms remain unclear. Firstly, temperature can change the workload of respiratory system, such as causing the excessive heat dissipation in the situation of high temperature [18]. This may be responsible for the differential effects of air pollution in different temperature conditions. Secondly, extreme temperature can elevate the workload of cardiovascular systems, such as the increase in blood viscosity and fibrinogen concentrations [19, 20]. This makes people more vulnerable to air pollution exposure in the situation of extreme temperature. Thirdly, the differential effects of air pollution in different climate conditions may partly result from the difference in measurement errors of air pollution exposure across climate conditions, because time spent outdoors and living habitat can vary among areas with different climatic conditions [6, 20].

Empirically, most are daily time-series studies that investigate the modification effect of air temperature. Generally, the findings from these time-series studies are quite mixed. Several studies suggest a larger effect of air pollution on health outcome in the situation of high air temperature [6, 21–23]. By contrast, some studies indicate the negative modification effect of air temperature [12, 19, 24]. However, few studies report the result that there is no modification effect of air temperature [25, 26].

Besides inconsistent findings above, further investigations are required owing to the following reasons. Firstly, few multi-site or nationwide studies have been conducted in developing countries. Of studies performed in developing settings, most are single-site examinations using time-series design [27–30]. This may partly result from the unavailable time-series data of health outcome at the nationwide level. Such single-site investigations may reduce the generalizability or potentially create the selection bias, which thereby is not sufficient to conclude the modification effect of air temperature. Despite the great necessity of nationwide studies, however, such studies are quite limited in developing countries, especially for China.

Secondly, few studies pay attention to air pollutants such as PM2.5 and PM1. Most studies place attention on PM10 and O3 [31–33]. By contrast, some air pollutants have been rarely focused, particularly for PM1 (particular matter with aerodynamic diameter < 1 μm). Increasing evidence has indicated that compared to the dominant PMs (e.g. PM2.5 and PM10) pollution in China, PM1 pollution has a larger effect on the health of human body [16, 17, 34, 35]. This is partly because the particle size of PM1 is smaller, which makes PM1 be inhaled into the deeper place of human lungs, thus causing a stronger effect on the physical health of human beings. However, few studies have focused on PM1 pollution, partly as a result of the limited or unavailable PM1 data in nationwide China.

Thirdly, little research, if at all, has investigated the modification effects of climatic factors. Most studies have examined the modification effect of daily air temperature [10, 30, 36, 37]. By contrast, few studies pay attention to climatic factors although most of these studies have reported the climatic modification effects [6, 38, 39]. Moreover, it has been suggested that the heterogeneous effects of environmental elements (e.g. air pollution and temperature) on human health, can be related to not only daily meteorological factors, but also the long-term climatic conditions such as the annual average temperature [5, 40, 41]. The proposed modification effects of climatic factors can be further supported by the seasonal and geographic differences in air pollution effects which have been observed in some previous studies [28, 42–44]. However, studies that examine climatic modification effects are still limited.

To fill the aforementioned gaps, this study aims to examine whether there are modification effects of climatic factors (i.e. air temperature and relative humidity) on the association between PM1 and the incidence rate of lung cancer using data collected from 345 Chinese cancer registries between 2014 and 2015. In terms of the stratified and combined datasets according to the three-category and binary divisions of climatic factors, we investigated climatic modification effects using the multivariable linear regression model adjusting for time, location, climatic conditions and socioeconomic covariates. We also performed three sensitivity analyses to test whether the findings of climatic factors with significant modification effects are robust when smoking factors are adjusted, and when data are stratified according to climatic factors over different periods or the different cut-offs (i.e. tertile division) of climatic factors.

## Materials and methods

### Research area

**Fig. 1:**
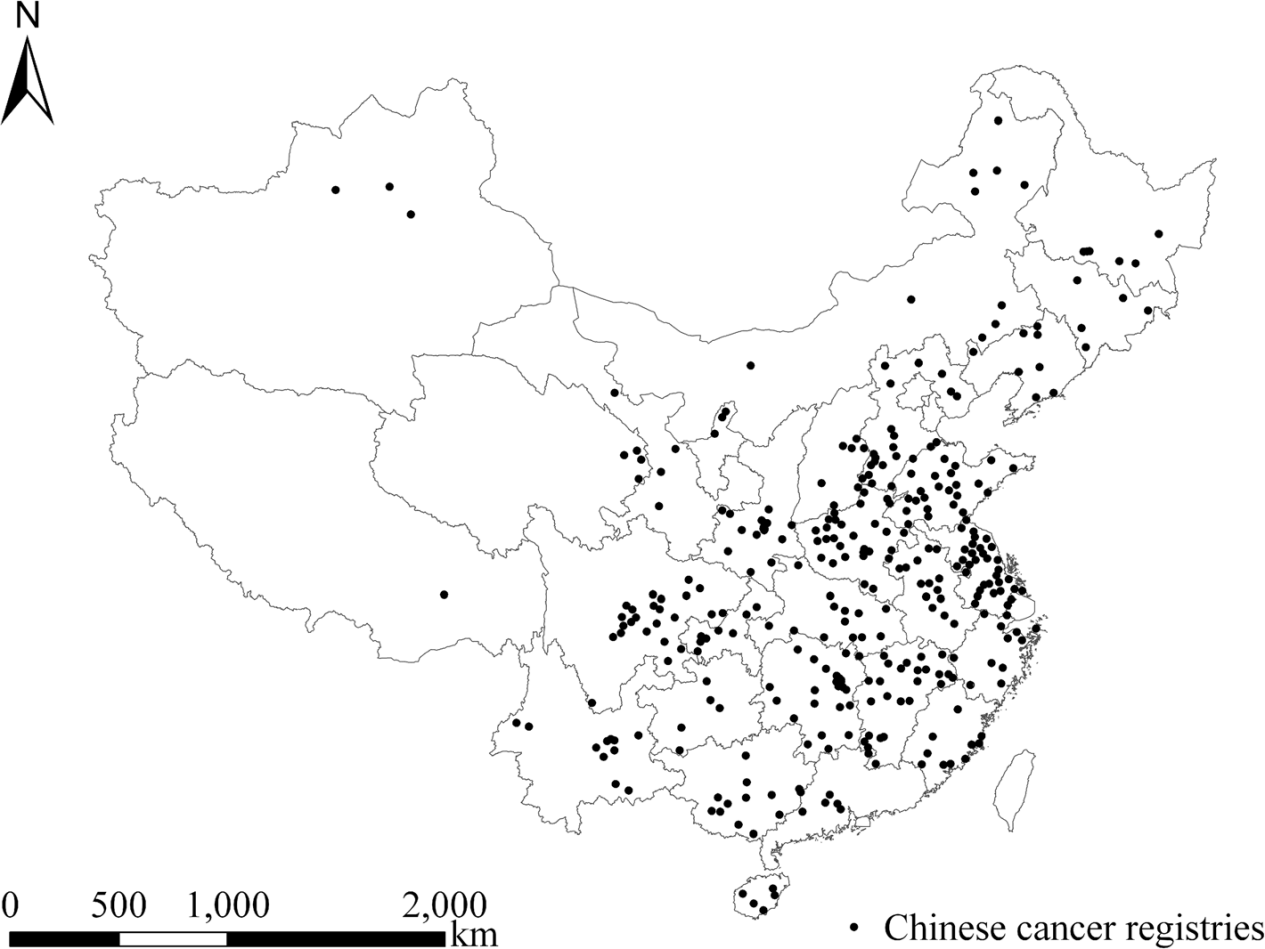
Spatial distributions of 345 Chinese cancer registries

This study aims to determine the modification effects of climatic factors in 345 Chinese cancer registries (Fig. 1). There are 259 rural (counties) and 86 urban (districts) registries. These registries were selected mainly based on the available lung cancer (2006–2015) and PM1 mass concentration (2014–2018) data. The 345 cancer registries selected (between 2014 and 2015) are dispersed over 31 of 34 Chinese provinces, autonomous regions and municipalities, with a population of around 221.59 million in 2015. The annual mean PM1 across registries was 45.12 μg/m^3^ in 2014, which is higher than the value in 2015 at 33.37 μg/m^3^.

### Data collection

#### PM1 pollution

The variable of air pollution is the annual mean PM1 mass concentration aggregated in each county (or district) for the period 2014-2015. Ambient particular matter (e.g. PM1, PM2.5 and PM10) has become one of the leading causes of lung cancer disease in the world [2, 45]. As stated in the introduction section, the effect of PM1 on human health is larger than those of two other prominent PM pollutants (PM2.5 and PM10) in Chinese cities [16, 17, 35]. Moreover, less is known about the impacts of the interactions between PM1 and climatic factors on human health in China. Therefore, we focus on PM1 in the present study.

PM1 data were acquired from our previous study [46] which estimated daily PM1 concentrations in 1 km^2^ grid cells from 2014 to 2018 in China. More details of PM1 estimates can refer to Wei et al. [46]. Briefly, a space-time extremely randomized trees (STET) model was used for the estimate of near-surface PM1 concentrations with model inputs of MAIAC AOD (i.e. aerosol optical depth), MEIC pollution emissions, meteorological factors, land use, topography, road, population, and the spatiotemporal information. Considering the spatiotemporal autocorrelations of PM1 concentrations, as in some prior studies [47–49], Wei et al. [46] incorporated such autocorrelations as the inputs of the STET model, which has greatly improved the accuracy of the estimate of PM1 concentrations.

**Fig. 2:**
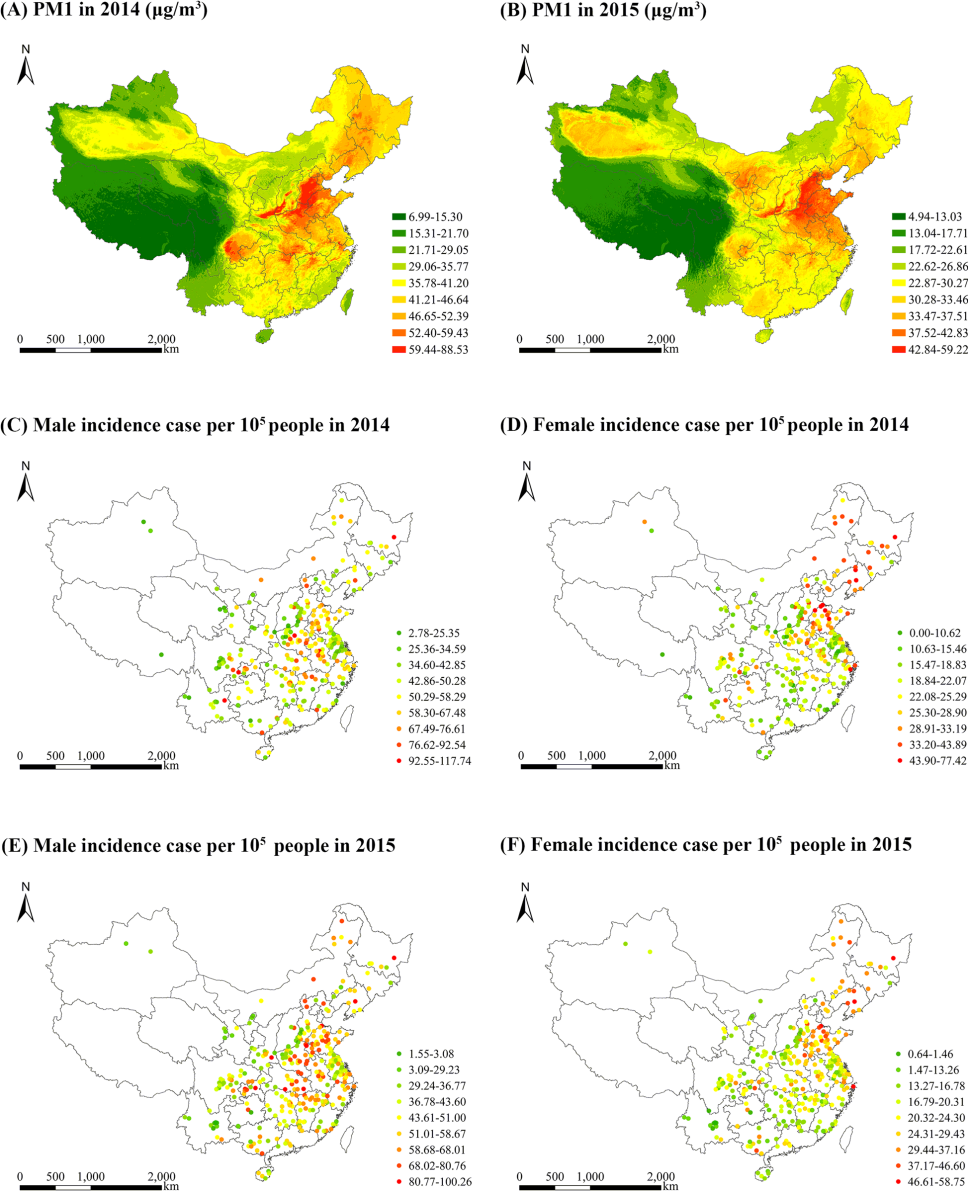
Spatial distributions of PM1 and the incidence rates for males and females in 2014 and 2015

In accordance with the results of ten-fold cross-validation, the STET-based daily PM1 estimates are highly in line with daily ground-level measurements with R^2^ and root-mean-square error (RMSE) equal to 0.77 and 14.6 μg/m^3^, respectively, which indicates good model performance [46]. The high consistency can also be observed at the seasonal and annual scale. That is, there is high consistency between the seasonal and annual PM1 estimates and ground-level measurements with R^2^ equal to 0.97 and RMSE less than 4.1 μg/m^3^. Figure 2 (a-b) present the spatial distributions of annual mean PM1 concentrations in 2014 and 2015, respectively.

#### Age-standardized incidence rate of lung cancer

Data on annual age-standardized incidence rates of lung cancer for males and females (i.e. the incidence rate of male (or female) lung cancer hereinafter) were collected from the 2017–2018 China Cancer Registry Annual Reports. The variable of health outcome is defined as the number of incidents of lung cancer for males (or females) per 100,000 people per year in a registry (e.g. county and district), which is age-standardised in accordance with the Segi’s world population. We place our attention on lung cancer, mainly because lung cancer has become the first-order cause of cancer incidences in China, with the age-standardized incidence rate of 36.54 per 100, 000 reported in the 2017 China Cancer Registry annual report [50].

In the annual reports, the causes of cancer incidences are specified in accordance with the International Classification of Diseases version 10 (i.e. ICD-10, lung cancer: C33–34). They are annually released by the Chinese Cancer Registry of the National Cancer Centre, China, which aims to provide the timely information on cancer diseases, such as the incidence rate of lung cancer. These reports are considerably comprehensive and representative at the national scale. For instance, the 2017 China Cancer Registry Annual Report released the data of cancer incidences for 339 cancer registries in 2014, which covers 31 of 34 provinces, autonomous regions and municipalities in China [50]. Figure 2 (c-f) show the spatial distributions of the incidence rates of lung cancer for males and females in 2014 and 2015.

#### Climatic factors

The climatic variables are the annual mean air temperature and relative humidity which are aggregated in each county (or district). We collected climatic data from the dataset of ecv-for-climate-change (i.e. essential climate variables for assessment of climate variability from 1979 to present), publicly released by the European Centre for Medium-Range Weather Forecasts (i.e. ECMWF) (https://cds.climate.copernicus.eu/cdsapp#!/dataset/ecv-for-climate-change?tab=overview). This dataset provides a monthly time-series data of climate variables (including surface air temperature and air relative humidity) at 25 km^2^ spatial resolution from 1979 to present. The ecv-for-climate-change dataset was drawn from the two datasets of the ECMWF, namely, ERA5 and ERA-Interim re-analyses.

In the ecv-for-climate-change dataset, data on monthly surface air temperature and relative humidity (except the data over the Great Lakes) were derived from the dataset of monthly mean ERA5 reanalysis. The ERA5 reanalysis dataset was generated using the 4D-Var data assimilation in Cycle 41R2 of Integrated Forecasting System (IFS) of ECMWF. According to the results of validation, the ERA5-derived air temperature is highly consistent with monitoring measurements in nationwide China, with the annual average RMS (i.e. Root Mean Square) equal to 2.31 °C [51]. Such high consistencies with ground observations have also been reported for several climatic variables derived from the ERA5 product [20, 52].

**Fig. 3:**
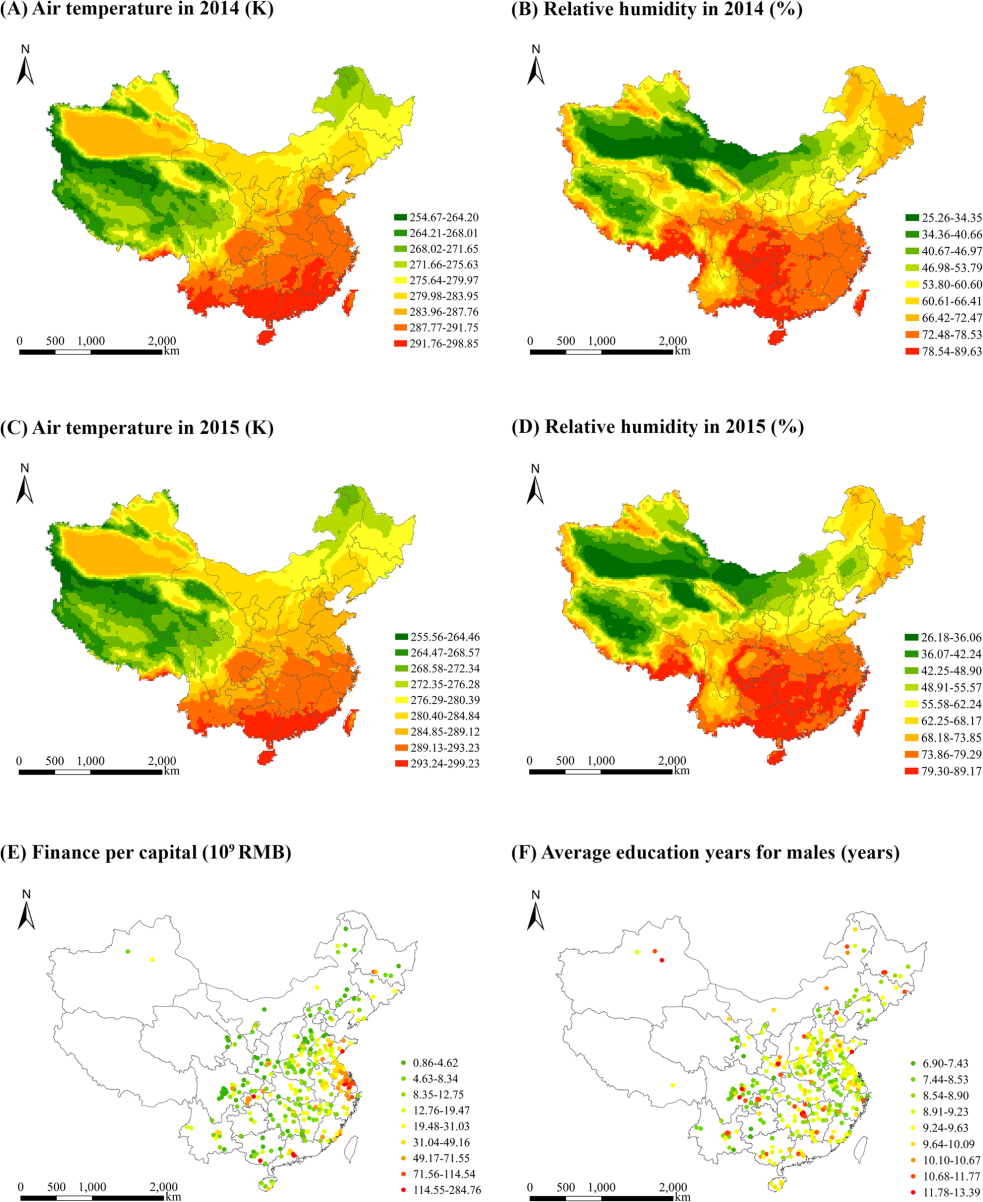
Spatial distributions of air temperature and relative humidity in 2014 and 2015

It has also been suggested that the ERA5 product outperforms prior reanalysis datasets in surface air temperature and humidity [53, 54]. Such better performance is in part because ERA5, the newest (fifth) generation of ECMWF global climate and weather analyses, has greatly improved its spatial and temporal resolutions for climatic data. To date, the ERA5 reanalysis dataset has been widely used in meteorological modelling [55] and the understanding of meteorological effects on air pollution, human health, and heat stress [56–58]. Figure 3 (a-d) present the spatial distributions of annual mean air temperature and relative humidity in 2014 and 2015.

#### Socioeconomic variables, location and time

Seven socioeconomic variables are used to control the difference in health outcomes associated with socioeconomic statuses. They include average education years, finance per capita, proportions of construction and manufacturing workers, urban–rural dummy variable, employment rate and population size. These socioeconomic factors are chosen to control the differential health outcomes (i.e. the incidence rate of lung cancer in the present study) in relation to economic status, educational attainment, occupation and socioeconomic statuses (i.e. a comprehensive measure). The socioeconomic data were derived from the China Statistical Yearbook (County level), Report on the Work of the Government, Tabulation of the 2010 Population Census of the People’s Republic of China, and the Statistical Communique on National Economic and Social Development. Figure 3 (e–f) illustrate the spatial distributions of some socioeconomic variables. In line with prior studies [9, 59, 60], we include longitude and latitude degrees and time dummy variable in the regression model to adjust for the effects of location and time, respectively.

#### Smoking factors

The variables of smoking include smoking prevalence and the number of cigarettes smoked per day, which have shown their associations with lung cancer diseases in previous studies [61]. The smoking data in 2015 were extracted from the module of health status and functioning of the 2015 China Health and Retirement Longitudinal Study (CHARLS) wave3, publicly released by the National School of Development of Peking University (http://charls.pku.edu.cn/en/page/data/2015-charls-wave4). As a high-quality representative survey across China, one of the aims of the CHARLS is to assess the health conditions of Chinese residents with ages 45 and older [62]. The CHARLS recruited 10,257 households and 17,708 individuals, which are located in 28 of 30 Chinese province-level administrative units (not include Tibet, Hong Kong, Taiwan and Macao) [62].

### Statistical analysis

The modification effects of climatic factors were determined in two stages. The first one was the stratification of dataset in accordance with climatic variables. The data were firstly stratified into three categories. Following previous studies [5, 6, 41], we used the mean air temperature and relative humidity over the study period (2014–2015) as the proxies of climatic conditions. Learning from prior research [3, 25, 63], the 25th and 75th percentiles were selected as the cut-offs of the two climatic factors to stratify data. Then, we further divided the data into two categories in terms of the binary divisions of air temperature and relative humidity. This is to examine the modification effects of climatic factors more robustly instead of the single three- or two-category division which is popular in most previous studies [12, 36, 37].

In the second stage, a multivariable linear regression model was developed to investigate the modification effects of climatic factors in the stratified and combined datasets. With respect to the examination in the stratified datasets, annual mean PM1 concentration, time dummy variable, longitude and latitude degrees, annual mean air temperature and relative humidity, average education years, finance per capita, proportions of construction and manufacturing workers, employment rate, urban–rural dummy variable and population size were included in the regression model to compare PM1 effects across climatic subgroups. Then, the stratified datasets were combined and the interaction between PM1 and climatic dummy variable was added to the regression model. We did not include climatic dummy variable in the model, mainly because of this variable’s high collinearities with PM1 and its interaction term (s).

Finally, we performed three sensitivity analyses. Firstly, we tested the sensitiveness of the modification effects of climatic factors to the control of smoking factors. Since the smoking data that we can obtain from the CHARLS survey are available at the level of prefectural city, we attributed districts/counties belonging to the same prefectural city with the same smoking characteristics. Moreover, the CHARLS survey where we extracted the smoking data does not cover all counties/districts of this study, so we kept the samples to counties/districts dispersed over the cities of the CHARLS survey, which left around 45% of the original number of registries (counties/districts) in such a  sensitivity analysis. To test the sensitiveness to the control of smoking covariates in a robust way, we performed such a test according to not only the original division (i.e. the original three-category and binary divisions which were based on the climatic factors of the original (total) number of counties/districts), but also the new division (i.e. the new three-category and binary divisions which were in terms of the climatic factors of the reduced number of counties/districts).

Secondly, we investigated whether the findings of climatic factors with significant modification effects are sensitive if data are stratified according to the different cut-offs (i.e. tertile division) of climatic factors, because some prior studies have reported the sensitiveness of modification effects to the different cut-offs [10, 32]. Thirdly, we tested whether the potential climatic modification effects are still robust when climatic factors over different periods (i.e. mean air temperature and relative humidity over a single year) are used as the further proxies of climatic conditions.

## Results

### Descriptive analysis

**Fig. 4:**
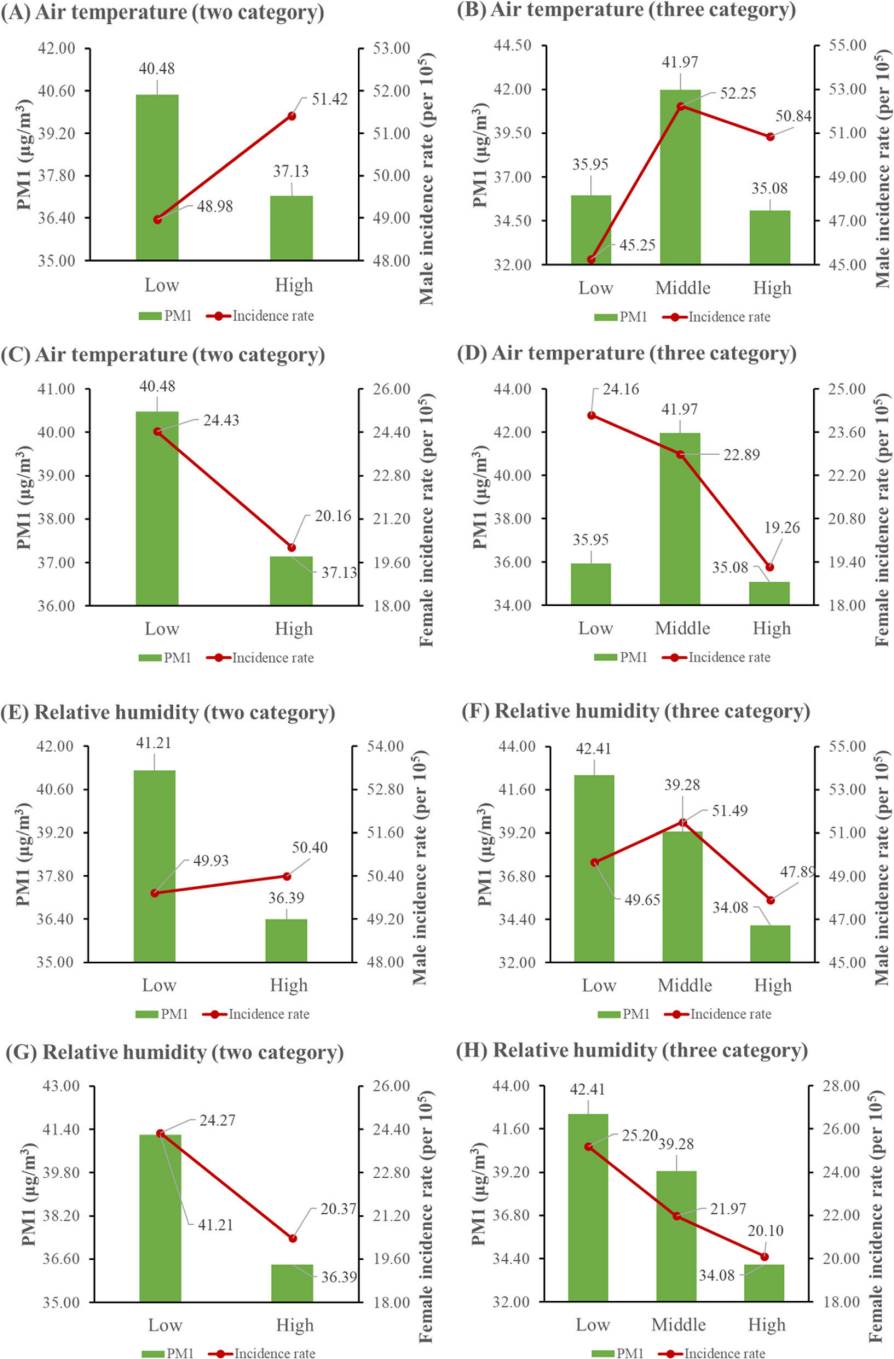
Descriptive statistics of PM1 and the incidence rate of male (or female) lung cancer in climatic stratums

Figure 4 shows the results of descriptive statistics of PM1 and the incidence rates of lung cancer for males and females in each climatic stratum. When data were stratified according to the binary division of temperature, the mean PM1 concentration in the low temperature group was 40.48 μg/m^3^, which is higher than the value of the high temperature group at 37.13 μg/m^3^ (Fig. 4 (a)). In contrast, there was a reversed pattern for the incidence of male lung cancer, with the values of 48.98 per 100,000 people and 51.42 per 100,000 people in the low and high temperature groups, respectively (Fig. 4 (a)). With regard to the binary division of relative humidity, the incidence rate of male lung cancer was higher in the high humidity group than in the low humidity group (Fig. 4 (e)); a contrary pattern of results was observed for the mean PM1 concentrations between the low and high humidity groups, with the values of 41.21 μg/m^3^ and 36.39 μg/m^3^, respectively (Fig. 4 (e)).

### Temperature modification effects

**Fig. 5:**
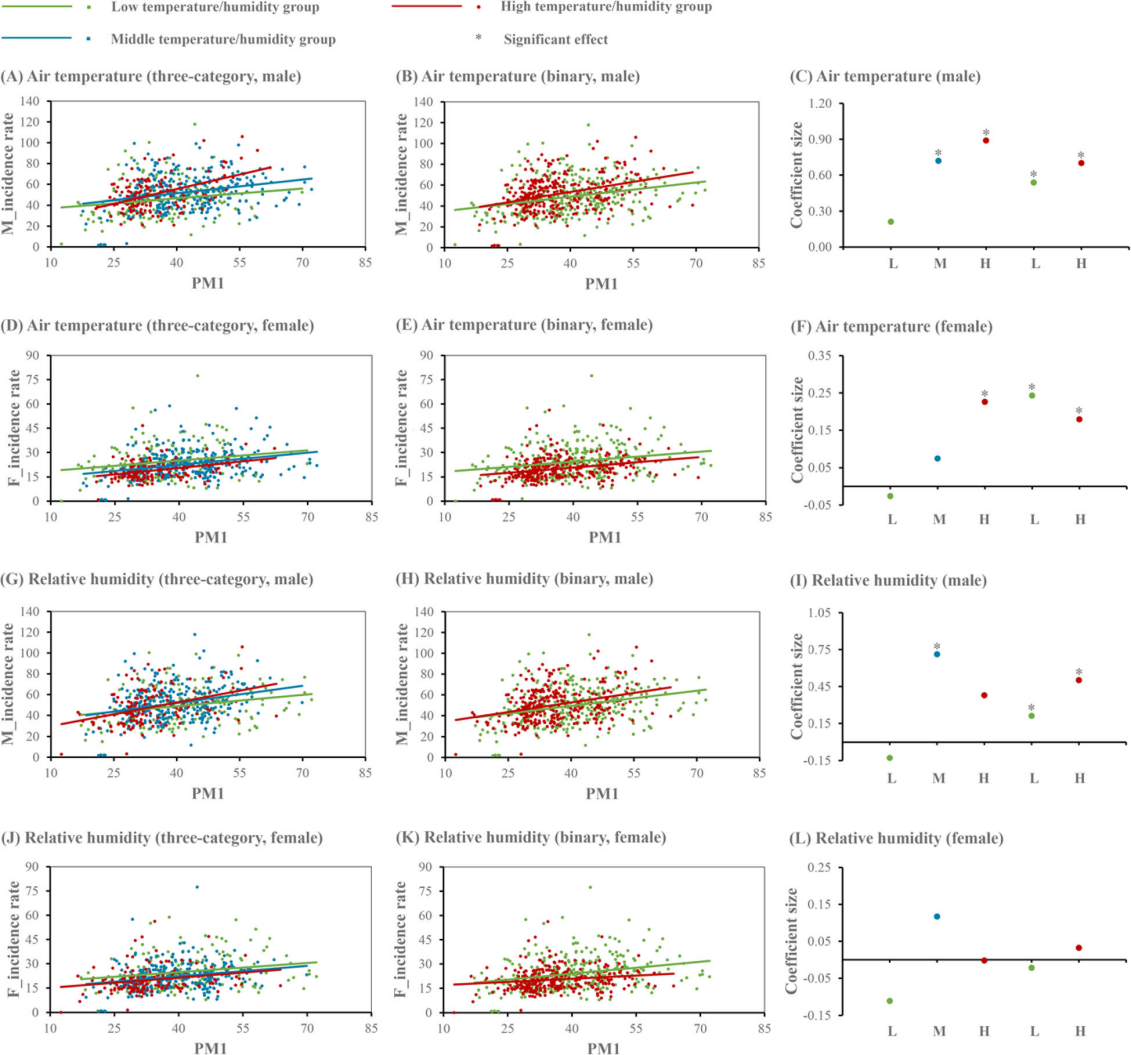
PM1 effects stratified by the three-category and binary divisions of climatic factors. M and F: male and female, respectively

Figure 5 (a-c) and Table 1 present the results of the modification effect of air temperature on the association between PM1 and male incidence rate. In general, air temperature positively modified such an association. Stratified datasets plotted in Fig. 5 (a-b) show that with the increase of air temperature, there was an increased effect of PM1 on the incidence rate of male lung cancer according to either the three-category or binary division of air temperature. In the stratified datasets of three-category division, a significant effect of PM1 was observed in the middle and high temperature groups but not in the low temperature group (Fig. 5 (c)). In the combined dataset of three-category division, if PM1 changes by 10 μg/m^3^, then the shift in male incidence rate relative to its mean was higher by 4.39% (95% CI: 2.19, 6.58%) and 8.37% (95% CI: 5.18, 11.56%) in the middle and high temperature groups than in the low temperature group, respectively (Table 1). With regard to the binary division, there was a significant effect of PM1 in the low and high temperature groups in the stratified datasets, with a smaller effect observed in the former group (Fig. 5 (c)); moreover, as shown in Table 1, the interaction between PM1 and temperature dummy variable was positively associated with the incidence rate of male lung cancer in the combined dataset (= 3.79, 95% CI: 1.99, 5.78%)

**Table 1:** PM1 and male incidence rate: Temperature modification effect

	Binary division		Three-category division	
	Mean male incidence rate = 50.16		Mean male incidence rate = 50.16	
	β	95% CI	β	95% CI
PM1	10.37% ***	(6.58%, 14.16%)	5.38%**	(0.40%, 10.17%)
Longitude	0.55 ***	(0.29, 0.80)	0.49 ***	(0.23, 0.75)
Latitude	0.20	(− 0.20, 0.61)	0.61***	(0.14, 1.07)
Year 2015	4.71 ***	(1.37, 8.04)	2.98 *	(− 0.45, 6.41)
Relative humidity	0.03	(−0.19, 0.26)	0.10	(− 0.12, 0.33)
Finance	0.04	(−0.03, 0.10)	0.03	(−0.03, 0.10)
Education	−1.94 *	(−4.13, 0.26)	−2.40 **	(−4.61, −0.19)
Employment	−0.20	(−0.44, 0.04)	−0.25 ***	(−0.49, − 0.01)
Construction	0.01	(−0.06, 0.08)	0.02	(−0.05, 0.09)
Manufacturing	−0.04 ***	(−0.07, − 0.01)	− 0.03 **	(− 0.06, − 0.01)
Population	0.02	(−0.03, 0.06)	0.01	(−0.04, 0.06)
Urban-rural division	3.18	(−1.02, 7.38)	3.03	(−1.14, 7.21)
PM1 × Temperature2	3.79% ***	(1.99%, 5.78%)	4.39% ***	(2.19%, 6.58%)
PM1 × Temperature3			8.37% ***	(5.18%, 11.56%)

* for *p* < 0.1, ** for *p* < 0.05 and *** for *p* < 0.01. If PM1 changes by 10 μg/m^3^, the change in incidence rate relative to its mean = (10 × coefficient for PM1 or its interaction terms)/mean incidence rate

The results of the modifying role of temperature on the association between PM1 and female incidence rate are shown in Fig. 5 (d-f) and Table 2. In general, there was no significant difference in PM1 effects among groups with different temperature levels. Figure 5 (d–e) plotted female incidence rate against PM1 concentration in each stratum according to the three-category and binary divisions of air temperature, respectively. With regard to the three-category division, the effect of PM1 was significant in the high temperature group but not in the low and middle temperature groups in the stratified datasets (Fig. 5 (f)); despite the positive effect of the interaction between PM1 and temperature dummy variable, the effect of PM1 was not statistically significant in the combined dataset (Table 2). Regarding the binary division, we did not observe the significant effect of PM1 in any temperature subgroup in the stratified datasets (Fig. 5 (f)); similarly, there was no significant effect of the interaction between PM1 and temperature dummy variable in the combined dataset (Table 2).

**Table 2:** PM1 and female incidence rate: Temperature modification effect

	Binary division		Three-category division	
	Mean female incidence rate = 22.37		Mean female incidence rate = 22.37	
	β	95% CI	β	95% CI
PM1	7.15% ***	(3.13%, 11.62%)	3.58%	(−2.24%, 8.94%)
Longitude	0.32 ***	(0.20, 0.45)	0.30 ***	(0.17, 0.43)
Latitude	0.55 ***	(0.35, 0.75)	0.72 ***	(0.49, 0.95)
Year 2015	0.40	(−1.23, 2.03)	−0.07	(−1.76, 1.61)
Relative humidity	0.07	(−0.04, 0.18)	0.07	(−0.04, 0.18)
Finance	0.06 ***	(0.02, 0.09)	0.05 ***	(0.02, 0.09)
Education	−1.50 ***	(−2.38, −0.61)	−1.48 ***	(−2.38, −0.58)
Employment	−0.03	(−0.15, 0.09)	−0.02	(−0.14, 0.10)
Construction	−0.05 ***	(−0.08, − 0.02)	− 0.05 ***	(− 0.08, − 0.02)
Manufacturing	−0.01 **	(−0.03, 0.00)	−0.01 **	(−0.03, 0.00)
Population	−0.02	(−0.04, 0.01)	−0.02	(−0.04, 0.00)
Urban-rural division	3.11 ***	(1.03, 5.19)	2.93 ***	(0.86, 5.00)
PM1 × Temperature2	0.89%	(−1.34%, 2.68%)	2.68% **	(0.45%, 5.36%)
PM1 × Temperature3			4.92% ***	(1.34%, 8.49%)

* for *p* < 0.1, ** for *p* < 0.05 and *** for *p* < 0.01. If PM1 changes by 10 μg/m^3^, the change in incidence rate relative to its mean = (10 × coefficient for PM1 or its interaction terms)/mean incidence rate

### Humidity modification effects

Relative humidity was positively related to the association between PM1 and male incidence rate. As plotted in Fig. 5 (g-h), an enlarged effect of PM1 on the incidence rate of male lung cancer was observed when there was an increase in the level of relative humidity. In the stratified datasets of three-category division, a significant effect of PM1 was observed in the middle humidity group but not in the low and high humidity groups (Fig. 5 (i)). In the combined dataset of three-category division, if there is a 10 μg/m^3^ change in PM1, then the change in male incidence rate relative to its mean was higher by 2.19% (95% CI: 0.40, 3.39%) and 3.59% (95% CI: 1.00, 5.98%) in the middle and high humidity groups compared with the low humidity group, respectively (Table 3). Regarding the binary division, we observed a significant effect of PM1 on male incidence rate in each humidity stratum in the stratified datasets, with a higher effect observed in the high humidity group (Fig. 5 (i)); moreover, the interaction between PM1 and humidity dummy variable was significantly associated with the incidence rate of male lung cancer in the combined dataset Table 3).

**Table 3:** PM1 and male incidence rate: Humidity modification effect

	Binary division		Three-category division	
	Mean male incidence rate = 50.16		Mean male incidence rate = 50.16	
	β	95% CI	β	95% CI
PM1	4.78% **	(0.20%, 9.57%)	4.59% **	(−0.20%, 9.37%)
Longitude	0.35 ***	(0.09, 0.62)	0.34 **	(0.06, 0.62)
Latitude	1.55 ***	(0.77, 2.32)	1.52 ***	(0.72, 2.32)
Year 2015	0.96	(−2.87, 4.78)	1.00	(− 2.85, 4.85)
Air temperature	1.79 ***	(0.95, 2.63)	1.88 ***	(1.02, 2.73)
Finance	0.04	(−0.03, 0.11)	0.04	(− 0.03, 0.10)
Education	−2.22 **	(−4.39, −0.04)	−2.05 **	(−4.24, 0.15)
Employment	−0.26 **	(−0.50, − 0.02)	− 0.30 **	(− 0.54, − 0.06)
Construction	0.01	(−0.05, 0.08)	0.01	(−0.06, 0.08)
Manufacturing	−0.04 ***	(−0.07, − 0.02)	− 0.03 **	(− 0.06, − 0.01)
Population	0.01	(−0.04, 0.05)	−0.01	(−0.06, 0.04)
Urban-rural division	3.65 *	(−0.50, 7.81)	2.65	(−1.50, 6.81)
PM1 × Humidity2	3.39% ***	(1.59%, 5.18%)	2.19% **	(0.40%, 3.39%)
PM1 × Humidity3			3.59% ***	(1.00%, 5.98%)

* for *p* < 0.1, ** for *p* < 0.05 and *** for *p* < 0.01. If PM1 changes by 10 μg/m^3^, the change in incidence rate relative to its mean = (10 × coefficient for PM1 or its interaction terms)/mean incidence rate

Figure 5 (j-l) and Table 4 present the results of the modification effect of relative humidity on the association between PM1 and female incidence rate. In general, we did not observe a significant modification effect of humidity on such an association. Figure 5 (j–k) plotted female incidence rate versus PM1 concentration in each stratum in accordance with humidity’s three-category and binary divisions, respectively. Regarding the three-category division, no significant effect of PM1 was observed in each humidity stratum in the stratified datasets (Fig. 5 (l)); only one of the interaction terms between PM1 and humidity dummy variable was significantly associated with female incidence rate in the combined dataset (Table 4). With respect to the binary division, we did not detect the significant effect of PM1 in the stratified datasets; likewise, the effect of the interaction between PM1 and humidity dummy variable was not statistically significant in the combined dataset

**Table 4:** PM1 and female incidence rate: Humidity modification effect

	Binary division		Three-category division	
	Mean female incidence rate = 22.37		Mean female incidence rate = 22.37	
	β	95% CI	β	95% CI
PM1	0.18%	(−4.92%, 5.36%)	−0.45%	(−5.36%, 4.92%)
Longitude	0.24 ***	(0.11, 0.37)	0.25 ***	(0.12, 0.39)
Latitude	1.22 ***	(0.84, 1.60)	1.32 ***	(0.93, 1.70)
Year 2015	−1.49	(−3.35, 0.37)	−1.62 **	(−3.46, 0.23)
Air temperature	0.87 ***	(0.46, 1.28)	0.94 ***	(0.53, 1.35)
Finance	0.06 ***	(0.03, 0.09)	0.06 ***	(0.02, 0.09)
Education	−1.54 ***	(−2.42, −0.67)	−1.60 ***	(−2.47, −0.73)
Employment	−0.05	(−0.17, 0.07)	−0.05	(−0.17, 0.06)
Construction	−0.05 ***	(−0.08, − 0.02)	− 0.05 ***	(− 0.08, − 0.02)
Manufacturing	−0.02 ***	(−0.03, 0.00)	−0.01 **	(−0.03, 0.00)
Population	−0.02 **	(−0.04, 0.00)	−0.02	(−0.04, 0.00)
Urban-rural division	2.89 ***	(0.85, 4.93)	2.96 ***	(0.95, 4.97)
PM1 × Humidity2	1.34%	(−0.45%, 3.58%)	0.00%	(−1.79%, 1.79%)
PM1 × Humidity3			4.02% ***	(1.34%, 6.71%)

* for *p* < 0.1, ** for *p* < 0.05 and *** for *p* < 0.01. If PM1 changes by 10 μg/m^3^, the change in incidence rate relative to its mean = (10 × coefficient for PM1 or its interaction terms)/mean incidence rate

### Sensitivity analysis

#### The control of smoking factors

**Fig. 6:**
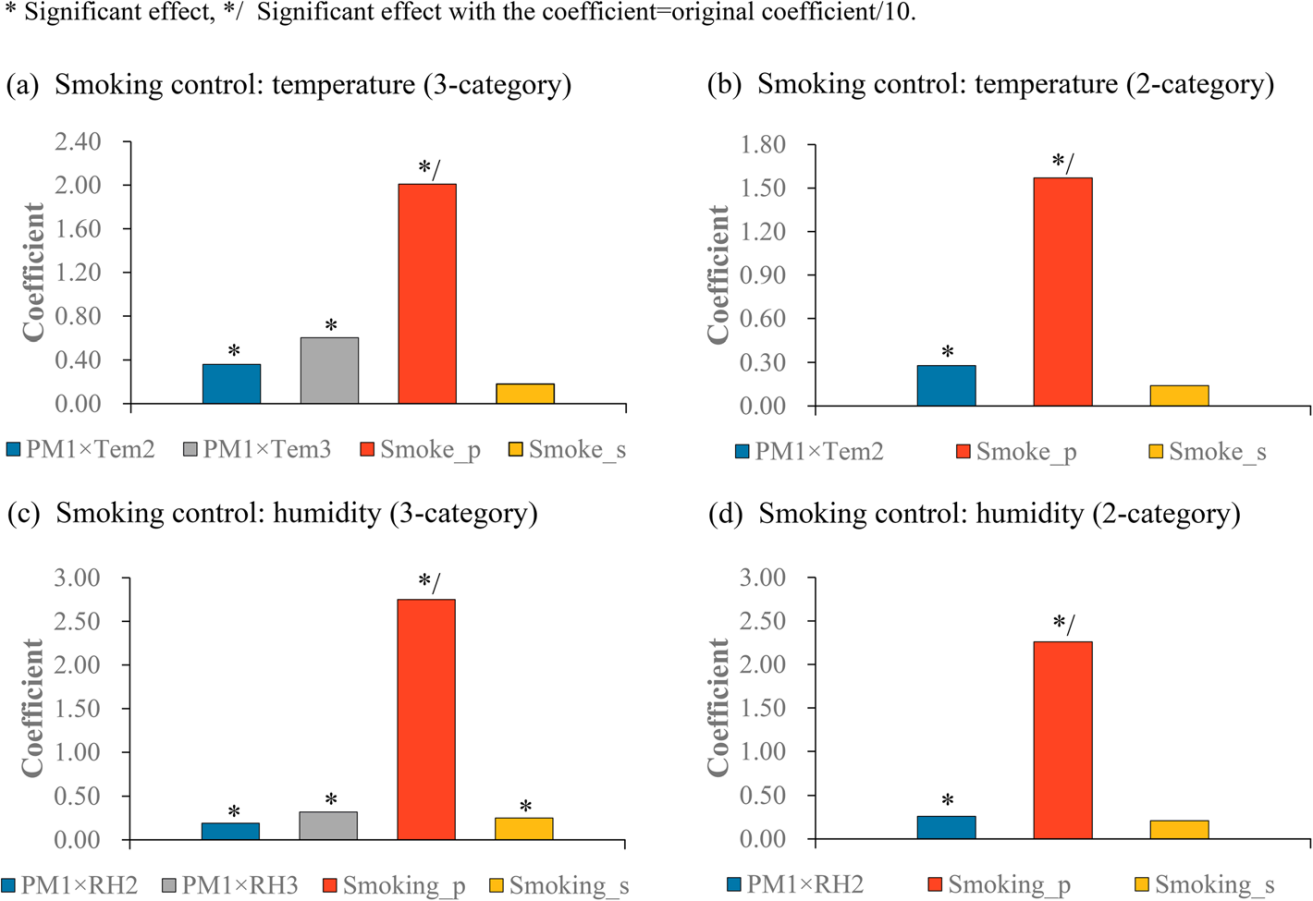
Climatic modification effects to the control of smoking factors (three-category and binary divisions were based on the climatic factors of 345 counties/districts). Tem means temperature; Smoke_p means smoking prevalence; Smoke_s means smoking strength

**Fig. 7:**
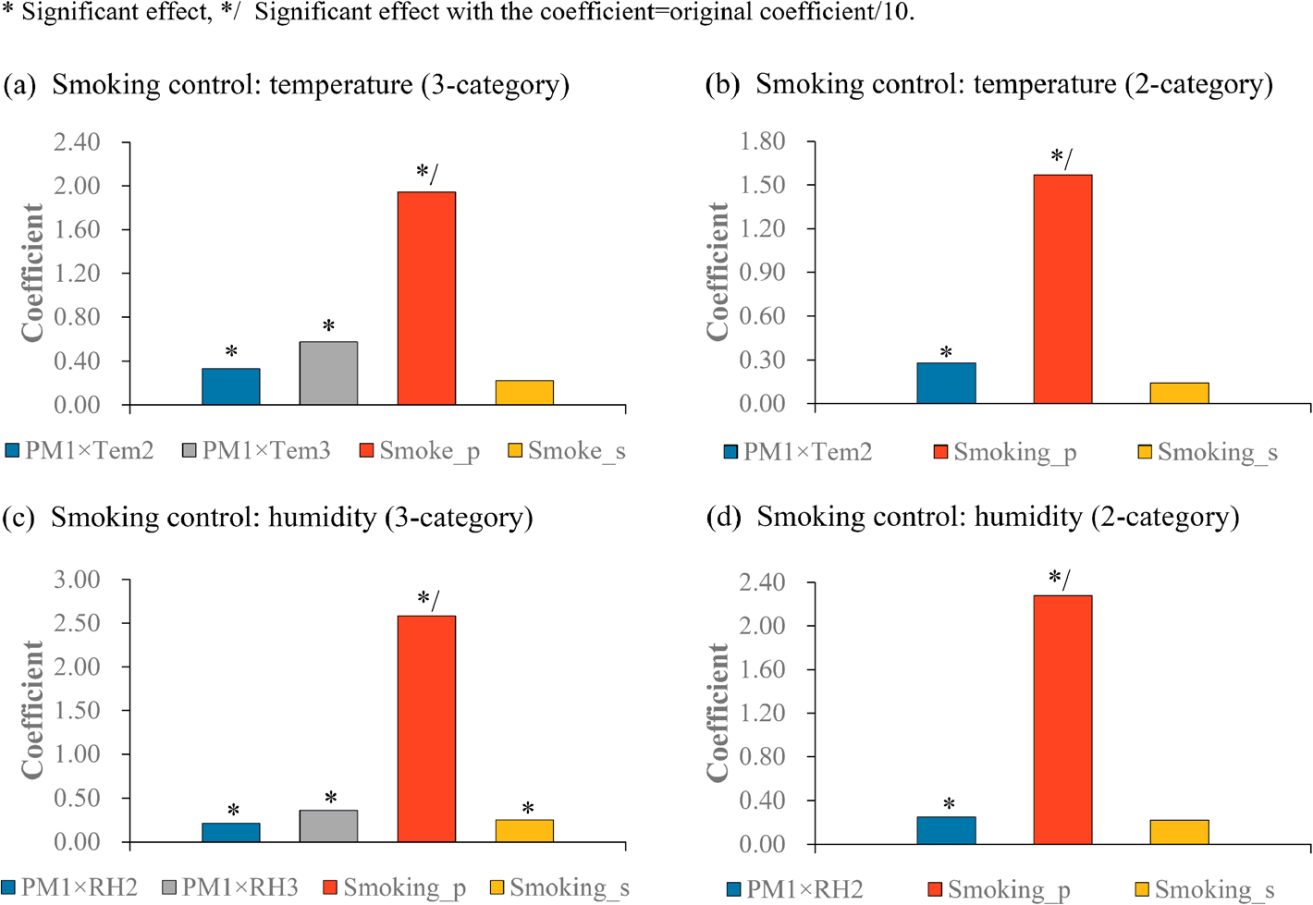
Climatic modification effects to the control of smoking factors (three-category and binary divisions were based on the climatic factors of the reduced counties/districts). Tem means temperature; Smoke_p means smoking prevalence; Smoke_s means smoking strength

Figures 6 and 7 show the results of the sensitivity analysis of climatic modification effects to the adjustment of smoking factors. Generally, the modification effects of temperature and humidity were robust to the control of smoking covariates. In the original division (i.e. the three-category and binary divisions were based on the climatic factors of the original (total) number of counties/districts), as shown in Fig. 6 (a), the interactions between PM1 and temperature dummy variable were positive in the combined dataset according to the three-category division of temperature. Likewise, smoking prevalence was positively associated with the incidence rate of male lung cancer (Fig. 6 (a)). Moreover, both smoking prevalence and the interaction between PM1 and temperature dummy variable were significantly correlated to the incidence rate of male lung cancer in accordance with the binary division of temperature (Fig. 6 (b)). A similar pattern of results was observed for humidity (Fig. 6 (c) and Fig. 6 (d)).

When the sensitiveness of climatic modification effects to the control of smoking covariates was tested using the new division (i.e. the three-category and binary divisions were based on the climatic factors of the reduced number of counties/districts) (Fig. 7), we observed that not only the interactions between PM1 and climatic factors but also some smoking factors had positive effects on the incidence rate of male lung cancer. In particular, as shown in Fig. 7(c), the significant effects of the interactions between PM1 and humidity dummy variable were observed; moreover, both smoking prevalence and smoking strength were positively associated with the incidence rate of male lung cancer.

#### Different cut-offs of climatic factors to stratify data

Table 5 presents the results of the sensitivity analysis using different cut-offs (i.e. tertile division) of climatic factors. In general, air temperature and relative humidity still positively modified the association between PM1 and male incidence rate when data were divided according to the tertile division. With respect to the tertile devision of air temperature, if there is a 10 μg/m^3^ shift in PM1, then the shift in male incidence rate relative to its mean was significantly higher by 3.59% (95% CI: 1.79, 5.58%) and 6.98% (95% CI: 4.39, 9.57%) in the middle and high temperature groups than in the low temperature group, respectively (Table 5). A similar pattern of results was observed for relative humidity. Specifically, the change in male incidence rate relative to its mean was higher by 1.50% (95% CI: − 0.32, 3.33%) and 3.75% (95% CI: 1.52, 5.99%) in the middle and high humidity groups compared with the low humidity group, respectively, when PM1 changed by 10 μg/m^3^ (Table 5).

**Table 5:** Different cut-offs of climatic factors to stratify data

	Air temperature			Relative humidity	
	Mean male incidence rate = 50.16			Mean male incidence rate = 50.16	
	β	95% CI		β	95% CI
PM1	7.18% ***	(2.79%, 11.36%)	PM1	4.71% **	(−0.05%, 9.48%)
Longitude	0.53 ***	(0.27, 0.78)	Longitude	0.36 ***	(0.09, 0.64)
Latitude	0.49 **	(0.05, 0.92)	Latitude	1.57 ***	(0.77, 2.37)
Year 2015	3.73 **	(0.39, 7.06)	Year 2015	0.97	(−2.88, 4.82)
Relative humidity	0.03	(−0.19, 0.26)	Air temperature	1.86 ***	(1.01, 2.71)
Finance	0.05	(−0.02, 0.11)	Finance	0.04	(−0.02, 0.11)
Education	−2.18 **	(−4.37, 0.01)	Education	−2.16 **	(−4.35, 0.02)
Employment	−0.21 *	(−0.45, 0.03)	Employment	−0.26 **	(−0.51, − 0.02)
Construction	0.03	(−0.04, 0.09)	Construction	0.01	(−0.06, 0.08)
Manufacturing	−0.04 ***	(−0.07, − 0.01)	Manufacturing	−0.04 ***	(−0.06, − 0.01)
Population	0.01	(−0.04, 0.05)	Population	0.00	(−0.05, 0.05)
Urban-rural	2.34	(−1.84, 6.52)	Urban-rural	3.43 *	(−0.73, 7.60)
PM1 × Temperature2	3.59% ***	(1.79%, 5.58%)	PM1 × Humidity2	1.50% *	(−0.32%, 3.33%)
PM1 × Temperature3	6.98% ***	(4.39%, 9.57%)	PM1 × Humidity3	3.75% ***	(1.52%, 5.99%)

* for *p* < 0.1, ** for *p* < 0.05 and *** for *p* < 0.01. If PM1 changes by 10 μg/m^3^, the change in incidence rate relative to its mean = (10 × coefficient for PM1 or its interaction terms)/mean incidence rate

#### Air temperature over a single year as a further proxy of climatic condition

Tables 6 and 7 show the results of the sensitivity analysis using climatic factors over different periods (i.e. over a single year) as further proxies of climatic conditions. In general, we still observed the positive modification effects of air temperature and relative humidity. As shown in Table 6, high temperature level was positively associated with the larger effect of PM1 on the incidence rate of male lung cancer according to either the three-category or binary division of air temperature. A similar pattern of results was observed for relative humidity (Table 6). Specifically, in the three-category division of relative humidity, if there is a 10 μg/m^3^ change in PM1, the change in male incidence rate relative to its mean was higher by 2.39% (95% CI: 0.60, 4.19%) and 2.99% (95% CI: 4.10, 5.78%) in the middle and high humidity groups than in the low humidity group, respectively (Table 7). In the binary division of relative humidity, the interaction between PM1 and humidity dummy variable was positively associated with the incidence rate of male lung cancer (= 3.59, 95% CI: 1.79, 5.38%).

**Table 6:** Air temperature over a single year as a further proxy of climatic condition

	Binary division		Three-category division	
	Mean male incidence rate = 50.16		Mean male incidence rate = 50.16	
	β	95% CI	β	95% CI
PM1	10.77% ***	(6.98%, 14.55%)	4.78% **	(0.00%, 9.77%)
Longitude	0.55 ***	(0.29, 0.81)	0.52 ***	(0.27, 0.78)
Latitude	0.13	(−0.28, 0.54)	0.62 ***	(0.17, 1.08)
Year 2015	4.72 ***	(1.36, 8.08)	2.97 *	(−0.43, 6.38)
Relative humidity	0.04	(−0.19, 0.27)	0.09	(−0.13, 0.31)
Finance	0.04	(−0.03, 0.10)	0.03	(−0.03, 0.10)
Education	−2.08 **	(−4.29, 0.13)	−2.22 **	(−4.40, −0.03)
Employment	−0.22 **	(−0.47, 0.02)	−0.24 **	(−0.48, 0.00)
Construction	0.01	(−0.06, 0.08)	0.02	(−0.04, 0.09)
Manufacturing	−0.04 ***	(−0.07, − 0.01)	− 0.04 ***	(− 0.06, − 0.01)
Population	0.02	(−0.03, 0.06)	0.01	(−0.04, 0.05)
Urban-rural division	3.45	(−0.77, 7.68)	3.06	(−1.09, 7.22)
PM1 × Temperature2	3.19% ***	(1.20%, 4.98%)	4.59% ***	(2.39%, 6.78%)
PM1 × Temperature3			8.97% ***	(5.98%, 12.16%)

* for *p* < 0.1, ** for *p* < 0.05 and *** for *p* < 0.01. If PM1 changes by 10 μg/m^3^, the change in incidence rate relative to its mean = (10 × coefficient for PM1 or its interaction terms)/mean incidence rate

**Table 7:** Relative humidity over a single year as a further proxy of climatic condition

	Binary division		Three-category division	
	Mean male incidence rate = 50.16		Mean male incidence rate = 50.16	
	β	95% CI	β	95% CI
PM1	4.78% **	(0.00%, 9.57%)	4.59% **	(−0.20%, 9.37%)
Longitude	0.33 **	(0.07, 0.60)	0.32 **	(0.05, 0.60)
Latitude	1.59 ***	(0.81, 2.37)	1.51 ***	(0.68, 2.34)
Year 2015	1.02	(−2.80, 4.84)	1.05	(−2.80, 4.89)
Air temperature	1.82 ***	(0.98, 2.65)	1.88 ***	(1.01, 2.75)
Finance	0.04	(−0.03, 0.10)	0.04	(−0.02, 0.11)
Education	−2.32 **	(−4.49, −0.14)	−2.16 **	(−4.36, 0.04)
Employment	−0.28 **	(−0.51, − 0.04)	− 0.30 **	(− 0.54, − 0.06)
Construction	0.01	(−0.05, 0.08)	0.01	(−0.06, 0.08)
Manufacturing	−0.04 ***	(−0.07, − 0.02)	− 0.03 ***	(− 0.06, − 0.01)
Population	0.01	(−0.04, 0.05)	−0.01	(−0.06, 0.04)
Urban-rural division	3.70 **	(−0.45, 7.85)	2.70	(−1.47, 6.86)
PM1 × Humidity2	3.59% ***	(1.79%, 5.38%)	2.39% ***	(0.60%, 4.19%)
PM1 × Humidity3			2.99% **	(0.40%, 5.78%)

* for *p* < 0.1, ** for *p* < 0.05 and *** for *p* < 0.01. If PM1 changes by 10 μg/m^3^, the change in incidence rate relative to its mean = (10 × coefficient for PM1 or its interaction terms)/mean incidence rate

## Discussion

### New insights from this study

A better understanding of climatic modification effects is essential to comprehend the differential effects and etiological pathways of air pollutions. However, it remains unknown whether climatic factors modify the effect of air pollution on the health of human beings. Moreover, nationwide studies that examine the modification effects of climatic factors are quite limited outside developed countries. Furthermore, few studies pay attention to PM1 despite its larger effect on human health.

To remedy these issues, we performed one of the earliest nationwide or multi-site studies [21] to investigate climatic modification effects on the association between PM1 and the incidence rate of lung cancer in China. This study contributes to the literature on climatic modification effects in a developing setting where air pollution is more severe than developed countries. We concentrate on PM1 pollution, which has its health effect larger than coarser PMs (e.g. PM2.5) but receives little attention.

We found a positive modification effect of air temperature (i.e. mean air temperature over the study period as a proxy of climatic condition) on the association between PM1 and the incidence rate of male lung cancer. This is consistent with that of some prior studies. In particular, collecting mortality data from the APHEA2 project (i.e. Air Pollution and Health: A European Approach 2), a multi-site study (i.e. 29 European cities) reported that the relative risk of daily death in relation to PM10 was 0.82% (95% CI: 0.69, 0.96%) in cities with warm climates, which is higher than the value of cites with cold climates at 0.29% (95% CI: 0.16, 0.42%) [6]. Likewise, a nationwide study of 207 American cities, which used mortality data derived from more than 35 million Medicare enrolees, indicated that the association between annual PM2.5 concentration and mortality was stronger in warm cities than in cold cities [41]. Evidence on the greater effect of air pollution observed in the situation of high temperature was further supported by the seasonal difference in air pollution effects reported in several previous studies [64–66].

Fundamental questions still remain how temperature modifies the effect of air pollution on human health. Currently, there is no agreement on the underlying mechanisms which are very complex. We speculate that the larger effect observed in the situation of high temperature may result from the difference in measurement errors of air pollution exposure [6, 20]. The ambient (or outdoor) measurement may represent individual exposure better in warmer counties than in colder counties, because residents in warmer counties are more likely to spend more time outdoors and keep their windows open [6, 20]. The second explanation may be that temperature can affect the chemical compositions of particulate matter. There are positive associations of temperature with sulfate, organic carbon, and elemental carbon [67]. Compared with other components of particular matter concentrations, sulfate and two types of carbon (organic and elemental) have greater effects on human health [68, 69]. Consequently, a high proportion of the sulfate and carbon components of PM mass, which is caused by high temperature, may be responsible for the greater effect of PM observed in the situation of high temperature.

We found that relative humidity (i.e. mean relative humidity over the study period as another proxy of climatic condition) positively modifies the effect of PM1 on the incidence rate of male lung cancer. That is, the effect of PM1 was smaller in the low humidity group than in the middle and high humidity groups. Despite some preliminary efforts [6, 70, 71], little is known about whether and how relative humidity modifies the association between air pollution and health outcome. More efforts are encouraged to determine the modification effect of relative humidity. We also found that the modification effect of air temperature is larger than that of relative humidity. In other words, air temperature is a more important effect modifier. This finding is in line with those reported from some previous studies [6]. In our study, if there is the same change in PM1 (i.e. 10 μg/m^3^ change in PM1), the change in the two interaction terms were 4.39% (95% CI: 2.19, 6.58%) and 8.37% (95% CI: 5.18, 11.56%) for air temperature, which are more than two times higher than the change in the two corresponding interaction terms at 2.19% (95% CI: 0.40, 3.39%) and 3.59% (95% CI: 1.00, 5.98%) for relative humidity. A similar pattern of results can be observed in accordance with the binary division of the two climatic factors.

### Strengths of the present study

There are some strengths in the present study. Firstly, we examine the modification effects of climatic factors on the long-term (instead of the short-term) association between air pollution and health outcome, which has been seldom investigated. Secondly, as an extension of most previous studies which solely focus on air temperature [3, 37, 43], we pay attention to the modifying roles of several climatic factors in the present study (i.e. air temperature and relative humidity as proxies of climatic condition). Thirdly, we concentrate on PM1 pollution, which has the largest health effect among the three prominent PM pollutants in China but receives quite little attention [16, 72]. Fourthly, most of previous studies place their attention on mortality [22, 27, 73], while we focus on incidence (or morbidity), which advances the understanding of climatic modification effects from a more comprehensive picture.

### Limitations and prospects

Several limitations and future work should be noted. Firstly, since county-level smoking data are not available in this study, we use the smoking data at city level to test the robustness of climatic modification effects to the control of smoking behavior. Such operation may ignore the variations in smoking characteristics among registries (i.e.counties/districts) located in the same city, which makes the sensitivity analysis suffer from the problem of ecological fallacy. Future work should handle such limitation if smoking data at county level are available. Moreover, despite the selection of smoking factors depends on the available smoking data, these selected smoking indicators (i.e. smoking prevalence and smoking strength) may not be sufficient to control the effect of smoking behavior. More smoking factors such as the age started smoking regularly and the prevalence of all smokers who used cigarettes should also be considered if data on these indicators are available in the future, which thus contributes to the robustness of the findings (climatic modification effects) observed in the present study.

Secondly, similar to most prior studies [10, 30, 41], there are potential errors in the estimate of PM1 exposure because mean PM1 concentration aggregated in each registry  (county/district) is used as the proxy of air pollution exposure in the present study. Thirdly, since our PM1 data are only available in 2014 and 2015, it is not feasible for us to test the sensitiveness of climatic modification effects to PM1 exposures with different lag structures (single and moving-average lags). Fourthly, given that most studies are ecological design in nature [6, 23, 25], studies using individual-level data to examine the modification effects of climatic factors are highly required in the future. The cross-sectional ecological examination is superior in not only its broader spatial coverage but also its large sample size. Cross-sectional ecological study, in combination with individual-level examination, can make great contributions to a more scientific and robust understanding of climatic modification effects. Finally, since most prior studies have investigated the modification effect of air temperature [3, 37, 43], future study can determine the modification effects of weather conditions (e.g. wind speed), season, and geographical factors.

## Conclusions

Climatic factors (i.e. air temperature and relative humidity as proxies of climatic condition) modify the association between PM1 and the incidence rate of male lung cancer in China. That is, males in counties with high levels of air temperature have high risks of PM1-associated lung cancer incidence in China; Chinese males living in counties with high relative humidity suffer from a greater effect of PM1 on lung cancer incidence. This study provides the evidence from a developing setting where air pollution is quite severe, which confirms the proposed hypothesis that climatic factors can modify the association between air pollution and the health of human beings in previous studies. Policy makers in public health should develop strategies that can simultaneously address air pollution and climate change. Such strategies can include the reduction of fossil fuel use and the development of a targeted warning system for air pollution and temperature, especially when air pollution and temperature are all at high levels. Future prediction or assessment on air pollution-associated health impacts should account for the climatic heterogeneity in air pollution effects.

## Supplementary Information

**Additional file 1: Table S1**. Descriptive statistics of socioeconomic factors and the incidence rate of lung cancer.

## Data Availability

The datasets during and/or analysed during the current study available from the corresponding author on reasonable request.
