# Supplementary material for: Climatic modification effects on the association between PM1 and lung cancer incidence in China
Source: BMC Public Health. 2021 May 7;21:880. doi: 10.1186/s12889-021-10912-8 (PMC8106137; doi:10.1186/s12889-021-10912-8)
Supplement: Supplementary file 1 — Additional file 1: Table S1. Descriptive statistics of socioeconomic factors and the incidence rate of lung cancer. [file 12889_2021_10912_MOESM1_ESM.docx]

Table S1. Descriptive statistics of socioeconomic factors and the incidence rate of lung cancer.

| Variables | Mean | SD | Min | Median | Max |
| --- | --- | --- | --- | --- | --- |
| Incidence rate of male lung cancer (per 10^5^ people) | 50.16 | 17.21 | 1.55 | 48.63 | 117.74 |
| Incidence rate of female lung cancer (per 10^5^ people) | 22.37 | 8.96 | 0.00 | 21.26 | 77.42 |
| Finance per capita (10^8^ RMB) | 23.04 | 29.58 | 0.81 | 13.18 | 284.76 |
| Population size (10^4^ people) | 64.48 | 34.59 | 4.00 | 59.00 | 186.23 |
| Average education year (year) | 9.35 | 1.02 | 6.90 | 9.12 | 13.39 |
| Manufacturing workers (%) ^a^ | 82.99 | 78.86 | 2.48 | 58.94 | 421.04 |
| Construction workers (%) ^a^ | 32.02 | 21.20 | 4.25 | 27.82 | 314.47 |
| Employment rate (%) | 69.71 | 8.23 | 40.92 | 71.29 | 86.15 |

^a^ for value = original value × 100.

Note: the descrpitive analysis for some indicators in Table S1 has been published in one of our previous paper with the paper information as follows:

Guo, H., Wei, J., Li, X., Ho, H. C., Song, Y., Wu, J., & Li, W. (2021). Do socioeconomic factors modify the effects of PM1 and SO2 on lung cancer incidence in China?. *Science of The Total Environment*,*756*, 143998.
